# Face shape processing *via* visual-to-auditory sensory substitution activates regions within the face processing networks in the absence of visual experience

**DOI:** 10.3389/fnins.2022.921321

**Published:** 2022-10-03

**Authors:** Roni Arbel, Benedetta Heimler, Amir Amedi

**Affiliations:** ^1^Department of Medical Neurobiology, Hadassah Ein-Kerem, Hebrew University of Jerusalem, Jerusalem, Israel; ^2^Faculty of Medicine, Hebrew University of Jerusalem, Jerusalem, Israel; ^3^Department of Pediatrics, Hadassah University Hospital-Mount Scopus, Jerusalem, Israel; ^4^Ivcher School of Psychology, The Institute for Brain, Mind, and Technology, Reichman University, Herzeliya, Israel; ^5^Center of Advanced Technologies in Rehabilitation, Sheba Medical Center, Ramat Gan, Israel

**Keywords:** fMRI, fusiform gyrus, visual deprivation, face perception, sensory substitution device, blindness, training

## Abstract

Previous evidence suggests that visual experience is crucial for the emergence and tuning of the typical neural system for face recognition. To challenge this conclusion, we trained congenitally blind adults to recognize faces *via* visual-to-auditory sensory-substitution (SDD). Our results showed a preference for trained faces over other SSD-conveyed visual categories in the fusiform gyrus and in other known face-responsive-regions of the deprived ventral visual stream. We also observed a parametric modulation in the same cortical regions, for face orientation (upright vs. inverted) and face novelty (trained vs. untrained). Our results strengthen the conclusion that there is a predisposition for sensory-independent and computation-specific processing in specific cortical regions that can be retained in life-long sensory deprivation, independently of previous perceptual experience. They also highlight that if the right training is provided, such cortical preference maintains its tuning to what were considered visual-specific face features.

## Introduction

Visual face processing is a complex visual skill that enables the identification of others, as well as the interpretation of expressions and affect. This skill develops through the exposure to hundreds of thousands of face exemplars over the years in varying conditions of light, changes in expression, gaze, and age (Maurer et al., 2002; McKone et al., 2007; Haist et al., 2013). A broad network of brain regions involved in visual face recognition has been identified using Functional Magnetic Resonance Imaging (fMRI). The Fusiform Face Area (FFA) is located in the visual extrastriate cortex and it is considered the hallmark of face cortical processing in the human brain. FFA has notable reproducibility for face identification and is part of the object-recognition system within the ventral visual stream (Kanwisher et al., 1997; Spiridon et al., 2006). This ventral-stream node has been shown to be strongly responsive to the invariant face components used for identity judgment when presented in the upright orientation (Haxby et al., 2000; Ishai, 2008) and to have a preference for familiar over unfamiliar face exemplars (Visconti di Oleggio Castello et al., 2017). Additional face-responsive regions include the occipital face area (OFA) and the temporal face area in the superior temporal sulcus (STS); these, along with the FFA, compose the core network for face identification (Haxby et al., 2000; Ishai, 2008). The extended network for face recognition includes the amygdala and insula, where emotional responses to faces are processed, the anterior temporal lobe, which mediates aspects of biological information, and the inferior frontal gyrus (IFG), where face-related semantic aspects are processed (Haxby et al., 2000, 2002; Ishai et al., 2005; Gobbini and Haxby, 2007; Ishai, 2008; Avidan et al., 2014).

Since experience with faces relies heavily on visual input, will face-responsive regions retain their preference and unique properties in cases of life-long visual deprivation? This question is particularly relevant when embedded within accumulating evidence documenting that in congenitally blind adults, nearly all the known regions in higher-order visual cortices which were heretofore considered “visual” can be activated by any sensory modality (e.g., by audition or touch rather than vision), while retaining their functional selectivity, for example, to objects in the lateral occipital complex (e.g., Amedi et al., 2007), to spatial localization in the middle-occipital gyrus (e.g., Collignon et al., 2011), to spatial layout in the parahippocampal area (Wolbers et al., 2011), to motion detection in MT+/V5 (e.g., Ricciardi et al., 2007), or to letters and number identification in visual word form area and in number form area, respectively (e.g., Reich et al., 2011; Striem-Amit et al., 2012; Abboud et al., 2015).

This evidence has given rise to a new theory of brain organization proposing that brain specializations are driven by specific sensory-independent computations rather than by sensory-specific processes as classically conceived (Heimler et al., 2015; Amedi et al., 2017).

However, cortical regions in the ventral visual stream normally responding to visual faces, with a special emphasis on FFA, are suggested to diverge from this theory, as evidence of FFA-preserved computational-selectivity in congenitally blind adults is inconsistent (Bi et al., 2016). Specifically, one line of research investigated whether FFA becomes responsive to human-emitted sounds when lacking visual experience across the lifespan and thus not developing typical face preference. Within this framework, one study reported FFA-like activations in congenitally blind adults in response to certain types of human-emitted sounds [e.g., chewing sounds; see van den Hurk et al., 2017, and a replication in Murty et al. (2020)]. However, other studies failed to show any FFA compatible activations for a more distinctive set of person-specific sounds, namely voices (Hölig et al., 2014; Dormal et al., 2018). Voices are a “special” human-related sound for people who are blind, as they represent the type of sensory information the blind rely on the most to identify people in their everyday lives. This lack of FFA-related activation for voices suggests that the computation of FFA may not specifically rely on identity judgments and may be more related to the components of human shapes (Konkle and Caramazza, 2013; Shultz and McCarthy, 2014; Bi et al., 2016).

In accordance with this latter consideration, another line of research investigated whether potential face preference in congenitally blind individuals may arise from haptic exploration of 3D face images. Despite social constraints limiting the extent of human identification *via* touch in people who are congenitally blind (Murty et al., 2020), they are still able to gain some experience of the general structure of faces from their own faces and those of their loved ones. This is evidenced by studies showing the ability of the congenitally blind to successfully classify 3D objects as belonging to the face category (Kilgour and Lederman, 2002; Murty et al., 2020). These behavioral results have been explained by suggesting that their extensive experience with touch for the purposes of object recognition, could result in connections between the somatosensory system and the object-identification regions within the ventral visual stream, therefore also including face regions (Bi et al., 2016). A recent study tested this hypothesis by asking congenitally blind adults to explore 3D faces *via* touch, while investigating face preferences within a pre-defined region in the deprived ventral visual stream (Murty et al., 2020). Results revealed preferential activation for 3D haptically explored faces over other categories in the blind in a location similar to that of the sighted FFA. This study suggested that in the absence of visual experience, some aspects of face preference in the ventral visual stream can arise through the haptic modality. This result, however, stands in contrast with a previous investigation which failed to report ventral stream activations in the congenitally blind for haptic face exploration (Pietrini et al., 2004).

All these results highlight some unexplored questions about the properties of face preference in congenitally blind adults. First, it is still unknown whether face preference in the deprived ventral visual stream is retained only for tactile processing or it extends to other spared sensory modalities, i.e., audition, namely a sensory-modality that people who are blind do not typically use for object recognition. Previous studies showed that congenitally blind adults can successfully perceive various object shapes (but not faces) *via* audition using visual-to-auditory Sensory Substitution Devices (Amedi et al., 2007; Striem-Amit and Amedi, 2014; Abboud et al., 2015). These SSDs transform visual information into audition using a specific algorithm that maintains core visual features such as objects’ shapes, locations, and even color (Meijer, 1992; Abboud et al., 2014). However, the little overall experience with face-shapes that blind acquire through touch may not be anchored enough in the deprived visual cortex to support a generalization to an entirely novel sensory-modality (i.e., SSD-conveyed auditory face-shapes). Second, and furthermore, other distinctive characteristics of face specificity within the ventral visual stream, such as the preference for upright over inverted faces (Yovel and Kanwisher, 2005; Rosenthal et al., 2016) or the difference between familiar and novel faces (Visconti di Oleggio Castello et al., 2017) remain unexplored in the visually deprived population. Finally, previous studies mainly focused on FFA activations alone, and therefore it is still unclear whether other regions of the face network become also activated by atypical sensory modalities.

To address these open issues, we trained a group of congenitally blind adults to perceive face shapes *via* audition using visual-to-auditory SSDs. Our blind participants were expert SSD users with dozens of hours of previous training with SSDs, albeit tailored to the perception of simpler objects. To teach participants the perception of the much more complicated colorful face soundscapes, we designed a ∼12 h training program specifically tailored to teach the perception of face shapes in their upright orientation, alongside additional similarly long training targeting other “visual” categories. Specifically, we trained our participants to perceive short words *via* SSD using a novel auditory SSD alphabet we developed (Arbel et al., 2020). This allowed us to explore the role of subordinate object identification associated with face exemplars, as well as experience with a novel category of stimuli (Gauthier and Tarr, 1997; Gauthier et al., 1999, 2000a).

We also trained our participants to use SSDs to recognize hand gestures to investigate the extent to which deprived cortical regions retain their preference for general animate objects in addition to face processing in the blind brain, as in the sighted brain (Peelen and Downing, 2005; Konkle and Caramazza, 2013; Kaiser et al., 2014; Shultz and McCarthy, 2014; Fisher and Freiwald, 2015; Thorat et al., 2019). Hand gestures, like faces, are stimuli for which congenitally blind adults have significantly lower perceptual experience through their remaining sensory-modalities during daily life compared to the sighted, with the exception of proprioceptive cues (Bi et al., 2016).

Finally, we tested the responses of congenitally blind adults to human voices, the stimulus congenitally blind individuals rely on the most across their lifespan to identify other people. With this additional control, we investigated whether general person-specific sound processing overlaps with putative face-shape related preferences in the congenitally blind brain or rather emerges in distinctive voice-specific regions (Gougoux et al., 2009; Dormal et al., 2018) (for results in the deaf brain, see Benetti et al., 2017).

After training, we presented our participants with stimuli belonging to all the above categories, while testing their neural responses using fMRI.

Taken together, our results show a maintained preference for face shapes in the ventral visual stream of the congenitally blind brain, with properties largely similar to those reported for the sighted brain. Specifically, we show preference for trained upright faces vs. words in a location near the sighted FFA, as well as a preference for faces over scramble faces in a similar location, accompanied by activations in a location near the sighted OFA, another core face region. In addition, we also observed a modulation by orientation and novelty of faces such that trained upright faces activated the face-responsive regions more than untrained inverted faces and of entirely novel faces. Crucially, both hand-gestures and faces activated the fusiform gyrus, albeit a Region of Interest (ROI) analysis suggests stronger activations for faces than hand-gestures. This latter result suggests that animacy processing is retained in the deprived fusiform gyrus. Finally, and in line with previous evidence, no activation for voices emerged in the deprived ventral visual stream. Taken together, these results further suggest that visual experience early in life is not the key factor shaping the emergence and properties of typical face preference in the ventral visual stream.

## Materials and methods

### Participants

Seven congenitally blind participants (five women, average age: 39 ± 5.3 years) with no reported neurological conditions or contra-indications for undergoing MRI scans, and with extensive (>50 h) experience with SSDs participated in the experiments. For detailed characteristics of participants see Table 1.

**TABLE 1:** Participants’ demographic information.

Participant	Age	Blindness cause	Light perception	Age at blindness onset	Braille reading	Handedness
FO	32	Microphthalmia	No	0	Yes (since age 5)	Right
FH	41	Leber’s disease	Faint	0	Yes (since age 5)	Ambidextrous
NN	44	Retinopathy of prematurity	No	0	Yes (since age 6)	Right
PC	40	Retinopathy of prematurity	No	0	Yes (since age 6)	Right
PH	41	Retinopathy of prematurity	No	0	Yes (since age 5)	Right
FN	32	Leber’s disease	Faint	0	Yes (since age 5)	Ambidextrous
DS	33	Retinopathy of prematurity	No	0	Yes (since age 6)	Right

The Hadassah Medical Center Ethics Committee approved the experimental procedure; written informed consent was obtained from each participant. Participants were reimbursed for their participation in the study.

### The EyeMusic algorithm

**FIGURE 1:**
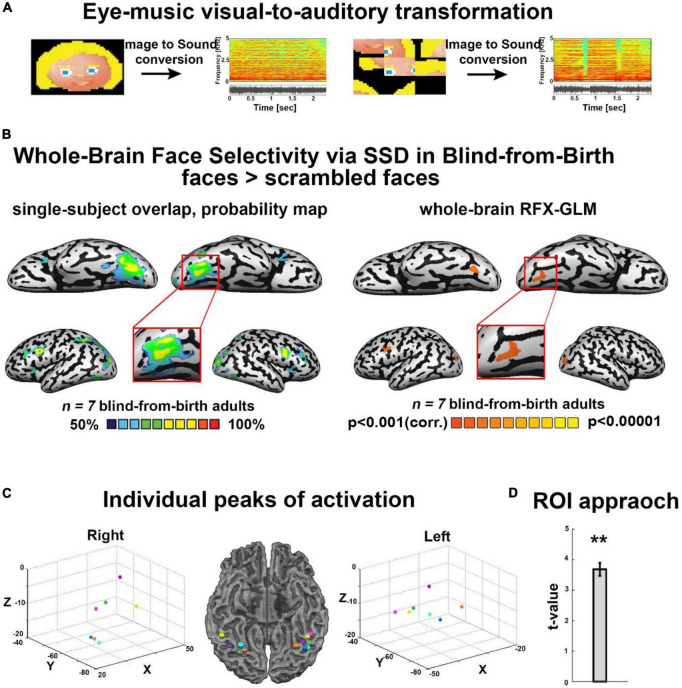
**(A)** Examples of some of the visual stimuli used, with their corresponding spectrograms after sonification *via* the Eye-Music visual-to-auditory SSD for trained and scrambled faces. **(B)** Trained faces > scrambled faces. Left: RFX-general linear model (GLM) analysis showed a bilateral cluster in fusiform gyrus (FG) (Talairach coordinates: right: 19, –68, –13 (zoom); left: –34, –65, –14). We also found bilateral clusters of activation in a region within the middle occipital gyrus, including the location of occipital face area (OFA) as described in the sighted (Talairach coordinates: right: 21, –94, –5; left: –33, –82, –8) and a cluster of activation in the left inferior frontal gyrus (IFG, Talairach coordinates: –45, 8, 31), two other cortical regions known to be involved in face processing. No preference for faces was observed in the auditory cortex (top panel). Right: The probability map obtained from the overlap of single participants’ activations for the same contrast reveals a remarkably consistent face preference in FG. **(C)** Trained faces > Scrambled faces. Individual peaks of activation in bilateral FG for all participants are located around the canonical location of the fusiform face area (FFA) in the sighted, lateral to the mid-fusiform sulcus. **(D)** Region of interest (ROI) approach: significant preference for trained faces > scrambled faces within the canonical location of the sighted right FFA. **P < 0.005.

The EyeMusic visual-to-auditory SSD was used to teach participants to identify whole-face shapes, as well as words and hand gestures (see details in the following section). EyeMusic transforms each pixel of a given image into what we term auditory soundscapes, namely an auditory pattern preserving shape, color, and spatial layout of objects. In brief, the EyeMusic algorithm down-samples each image to 50*30 pixels. Then, using a sweep-line approach, it transforms each pixel in a given image into a corresponding sound using the following parameters. First, the *x*-axis is mapped to time; each image is scanned column-by-column from left to right, such that pixels on the left side of the image are played before those on the right. Second, the *y*-axis is mapped to pitch variations using the pentatonic scale, such that the lower the pixel in the image, the lower the corresponding pitch sonifying it. Third, color is conveyed through timbre manipulations, such that each color is played using a different musical instrument, and brightness levels are conveyed *via* sound volume variations. EyeMusic has five colors (white, green, red, blue, and yellow), and black is mapped as silence (Figure 1A) (for full details, see Abboud et al., 2014).

### EyeMusic training sessions

#### Face identification

Face identification *via* shape recognition is a visually dominant skill with which congenitally blind adults have little if any experience. Therefore, before training, our congenitally blind participants were largely unaware of face-related shapes. Thus, a crucial aspect of our structured training program was familiarization with this novel object category.

Furthermore, auditory face identification is an extremely challenging behavioral task because of the complexity of the soundscapes created by faces. Therefore, our training program also focused on teaching participants how to interpret such auditory complex soundscapes. This is why we selected participants who were already proficient EyeMusic users with extensive previous training with the device (>50 h) before face training began. Note, however, that unlike their previous training which consisted mostly of line drawing of simple shapes, participants were presented with images filled with color and with colorful features embedded within the images whose interpretation was crucial to succeed in the task.

We constructed a training program that included five sessions of 2 h each (participants received additional “refresher” sessions up to 12 h in total). For full details of the training program see Arbel et al., 2022. In brief, participants were introduced to 6 cartoon faces which were adapted from the children’s game “guess who” and translated into soundscapes using EyeMusic (see Supplementary Figure 2A). In the first stages of the training, participants learned to interpret only horizontal strips of the images (bottom, top, and middle), to gradually advance their skill so they could focus on perceiving small details embedded within the complex sounds (Supplementary Figure 1). Even if during this phase we presented portions of faces, we never trained single facial features in isolation: each strip contained multiple facial features (e.g., each top strip contained hair, eyes, glasses, etc.). This is very different from tactile exploration where each facial feature is generally explored alone, separately from the others.

After familiarization with face-strips, participants were gradually introduced to the full face images, until they learned to identify all 6 cartoon faces. Identification consisted of learning the perception of each of these faces as a whole, as well as the features included in each face (e.g., eye or hair color, glasses, beard, etc.), and only then learning the name associated with the face. This latter training strategy was introduced because it was shown that assigning names to new faces improves face recognition skills (Schwartz and Yovel, 2016). In addition, it was introduced to make the training program more similar to our other training programs (see below) where each trained object had distinctive names.

#### Word training and other visual category training

The word training aimed at teaching our participants a novel orthography that we created by merging Braille and Morse features and transformed *via* EyeMusic to soundscapes (for full details see Arbel et al., 2020). During this structured training program, participants learned to read using this new orthography. The duration of training was the same as for face training. Training also included color, as each letter was consistently presented in one of three possible colors. Specifically, during training, participants first learned to identify half of the Hebrew alphabet (11 letters), and then learned to read short words and pseudo-words of up to five characters comprised of these letters. In the experiments described in this work, we included three 3-letter words using only trained letters.

Participants were trained to identify stimuli belonging to additional visual categories in similar computation-specific training programs. These included hand gestures (e.g., closed hands in a fist, fully open hands, closed fist with index, and middle finger stretched out – the three hand-gestures used in the game “rock, paper, and scissors”). A detailed description and results of these additional training programs will be reported more thoroughly in future publications.

### Behavioral experiments

Prior to the fMRI experiment, to ensure training effectiveness and the feasibility of the tasks inside the scanner, each participant completed three behavioral tasks [results are detailed in Arbel et al. (2022)]. Briefly, the first was a *naming task* in which participants named each of the six characters learned during training. Each character was played repeatedly until participant named it (roughly within two repetitions). Each character was presented in 16 separate trials, 96 trials overall presented in random order. Each face soundscape lasted 2.5 s, the same length as the soundscape presentations in the scanner. Participants provided their responses verbally, and the experimenter entered them into the computer. The rate of correct responses was analyzed using a *t*-test against chance level (17%) (see Supplementary Figure 2B).

The second task was an *orientation task*. A character was repeatedly played in either the standard upright (trained) or inverted (up-side down) orientation (untrained) until participants identified the orientation (roughly within two repetitions). Participants were instructed to verbalize their answer, and the experimenter entered the results into the computer. Each character in each orientation was presented five times (five separate trials) for a total of 60 trials presented in random order. Each face soundscape lasted 2.5 s, the same length as the soundscape presentations in the scanner. The rate of correct responses was analyzed, using a *t*-test against chance level (50%) (see Supplementary Figure 2A).

The third task was a *new-faces task*. Each of the six trained faces, as well as each of the six untrained faces, was presented repeatedly until participants identified whether the character was familiar or unfamiliar. Untrained faces were created using visual attributes similar to those of the trained faces (see Supplementary Figure 2A). Participants were instructed to verbalize their response, and the experimenter entered it into the computer. Each character was presented six times (six separate trials) for a total of 72 trials presented in random order. Each face soundscape lasted 2.5 s, the same length as the soundscape presented in the scanner. The rate of correct responses was analyzed (see Supplementary Figure 2B) using a *t*-test against chance level (50%).

The order of tasks was always the same: first, the naming task; then, the orientation task, and finally, the new-faces task following the fMRI session, to avoid presentation of the novel faces prior to investigation of neural processes mediating face perception. All behavioral tasks were programmed with the Presentation software.

### Functional magnetic resonance imaging experiments

#### Functional and anatomical magnetic resonance imaging acquisition

BOLD functional magnetic resonance imaging measurements were obtained in a whole-body, 3–T Magnetom Skyra scanner (Siemens, Germany). Scanning sessions included anatomical and functional imaging. Functional protocols were based on multi-slice gradient echoplanar imaging (EPI) and a 20 channel head coil. The functional data were collected under the following timing parameters: TR = 2 s, TE = 30 ms, FA = 70°, imaging matrix = 80 × 80, field of view (FOV) = 24 × 24 cm^2^ (i.e., in-plane resolution of 3 mm). Twenty-nine slices with slice thickness = 4 mm and 0.4 mm gap were oriented −22° from the axial position, for complete coverage of the whole cortex while minimizing artifacts from the frontal sinus. The first 10 images (during the first baseline rest condition) were excluded from the analysis because of non-steady state magnetization.

High resolution three-dimensional anatomical volumes were collected using a 3D-turbo field echo (TFE) T1-weighted sequence (equivalent to MP-RAGE). Typical parameters were: FOV 23 cm (RL) x 23 cm (VD) x 17 cm (AP); Foldover- axis: RL, data matrix: 160 × 160 × 144 zero-filled to 256 in all directions (approx. 1 mm isovoxel native data), TR/TE = 2,300 ms/2.98 ms, flip angle = 9°.

#### Pre-processing functional magnetic resonance imaging data

Data analysis was performed using Brain Voyager QX 2.0.8 software package (Brain Innovation, Maastricht, Netherlands). fMRI data pre-processing steps included head motion correction, slice scan time correction, and high-pass filtering (cut-off frequency: 2 cycles/scan). No head movement beyond 2 mm was detected in the collected data; thus, all participants were included in the subsequent analyses. Functional data underwent spatial smoothing (spatial Gaussian smoothing, full width at half maximum = 6 mm) to overcome inter-subject anatomical variability within and across experiments. Functional and anatomical datasets for each subject were first aligned (co-registered) and then transformed to fit the standardized Talairach space (Talairach and Tournoux, 1988).

#### Functional imaging experiments

##### Block-design experiment, modulated faces

First, to isolate the neural network recruited for auditory (Eye Music SSD) face recognition, we conducted a block-design experiment using four sets of face stimuli: condition a, *trained faces*; condition b, *trained faces in the untrained inverted orientation* (inverted faces); condition c, *entirely new, untrained faces* (new faces); condition d, *scrambled faces*. Condition a, trained faces, comprised six colorful faces participants learned to identify during training. In condition b, inverted faces, each of the six trained faces was sonified using EyeMusic in its untrained, inverted (upside down) orientation. In condition c, new faces, six visually similar faces that were not introduced during training were presented. In condition d, scrambled faces, the six familiar faces were divided into nine parts, and then scrambled randomly using MATLAB. The resulting images were sonified *via* EyeMusic (see Supplementary Figure 2A for visual representations of all stimuli, as well as spectrograms of the soundscapes in the experiment).

The conditions were presented in a block design paradigm. The experiment was programmed using Presentation software. Each condition was repeated 6 times, in a pseudorandom order, for a total of 24 blocks. To increase data robustness, we collected data over 4 runs using the following design. In each block, two different stimuli belonging to the same experimental condition were displayed, each lasting 5 s (two consecutive repetitions of 2.5 s per stimulus), followed by a response interval of 2 s. Each block started with an auditory cue indicating the tested category which lasted for 2 s (trained faces, inverted, new, and scrambled). Participants were instructed to listen carefully to the soundscapes and to provide their responses using a response box at the end of both repetitions of a stimulus. Motor tasks were added to confirm participants remained engaged during the entire length of the experiment. For trained and inverted conditions, participants were instructed to identify the character. To limit the number of stimuli of each condition to three and thus allow only one response box in the scanner, two of the four runs consisted of female characters in all blocks presented, and two runs consisted of male characters in all blocks presented. Each block lasted 16 s and was followed by a 10 s rest interval. New faces could not be asked to be directly identified, as they were introduced to participants for the first time during training. Thus, participants were instructed to listen carefully to each soundscape; to control for motor response and to ensure that participants paid attention to the shape characteristics of the presented soundscapes, we used a vision-related task: if they could identify yellow features, they were instructed to press 1, if not, 2. For scrambled faces, participants were instructed to closely attend to the auditory stimuli. Additionally, to ensure engagement and attendance to these stimuli, as well as a motor control, they were also instructed to press any response key at the end of each stimulus presentation.

Before entering the scanner, participants were familiarized with the tasks inside the scanner, especially concerning the response box, to make sure the response mapping was fully understood by all. Digital auditory soundscapes were generated on a PC, played on a stereo system, and transferred binaurally to the subjects through a pneumatic device and silicone tubes into commercially available noise shielding headphones.

##### Visual categories experiments

###### Event-related experiment

Stimuli from three categories were included. Faces: soundscapes of the three male trained characters. Written words: soundscapes of three 3-letter words presented in a novel auditory orthography designed for EyeMusic compatibility. Hand gestures: soundscapes of three trained hand gestures were included in the experiment, taken from the paper-rock-scissors game: a hand featuring the gesture of a “rock” (fist), a gesture of “paper” (open hand), and “scissors” (two straight fingers, other fingers in a fist). The conditions were presented in an event-related paradigm. The experiment was programmed using Presentation software. Each trial consisted of two repetitions of a stimulus, lasting 2.5 s each, followed by a rest interval of 11 s.

Participants were instructed to covertly classify each of the stimuli as belonging to the face, words or hand-gesture category and to identify each exemplar (i.e., identify the specific face character, read the specific word or identify the specific hand-gesture presented). The experiment consisted of two identical runs. Over the two runs, each stimulus was repeated 10 times, for a total of 30 trials per category.

###### Block design experiment

The same nine stimuli from the above experiment were included, together with three soundscapes of scrambled images of houses as control stimuli, presented in a block design. The experiment was programmed using Presentation software.

In each block, two different stimuli of the same condition were presented, each lasting 5 s (two consecutive repetitions of 2.5 s per stimulus), followed by a response interval of 2 s. Each block started with an auditory cue indicating tested category (faces, words, hand gestures) lasting 2 s. All blocks lasted 16 s and were followed by a 10 s rest interval. Participants were instructed to identify each stimulus and provide their responses using a response box after listening carefully. Before entering the scanner, all participants were familiarized with the stimulus-finger mapping. For the “scrambled” condition participants were instructed to press randomly, for a motor response.

Data were pooled for analysis across block and event-related designs for the “visual categories” experiments to increase the data’s robustness.

##### Human voice localizer

To localize the temporal-voice area (TVA) in our congenitally blind participants and explore possible ventral stream activations in response to human voices, we used the seminal fMRI voice localizer introduced by Belin (for details, see Belin et al., 2000; Pernet et al., 2015). In brief, the 10 min localizer contains blocks of vocal and non-vocal sounds in similar power spectra, allowing direct comparison of the two categories while avoiding auditory sampling bias. Vocal blocks contain human vocal sounds from different speakers, speech sounds and non-speech sounds (i.e., emotional and neutral sounds, such a cough). Non-vocal sounds contain natural sounds (e.g., wind), animals (e.g., sea waves), man-made sources (e.g., cars), and classical musical instruments (e.g., bells, harps). Each block lasted 20 s with 10 s of silence between consecutive blocks.

##### Quantification and statistical analysis

###### Probability mapping

To probe the anatomical consistency of face-specific regions among blind participants, we used overlap probability mapping. For each participant, we obtained individual activation maps for the specific contrast (Face > Scramble; Voice > Natural sounds; Faces > Baseline), with a threshold of *p* < 0.001 before correction, then corrected for multiple comparison using the spatial extent method based on the theory of Gaussian random fields (Friston et al., 1994; Forman et al., 1995). This was done based on the Monte Carlo stimulation approach, extended to 3D datasets using the threshold size plug-in for BrainVoyager QX with *p* < 0.05. Then, we computed the overlap probability. This was a way to investigate the consistency of activations across participants, as this analysis provided the percentage of participants who showed a given active voxel in the specific contrast of interest.

###### Whole-brain general linear model analysis

To compute statistical maps, we applied a general linear model (GLM) using predictors convoluted with a typical hemodynamic response function (Friston et al., 1999). Across-subject statistical maps were calculated using hierarchical random-effects model analysis (Friston et al., 1999). All GLM contrasts between two or more conditions included an additional comparison of the first conditions of the subtraction to baseline (rest times between the epochs), to ensure only positive BOLD changes would be included in the analysis. The minimum significance level of all results obtained using GLM analysis was set to *p* < 0.001 before correction, and then corrected for multiple comparisons to *p* < 0.05 using a cluster-size threshold adjustment for Monte Carlo simulation approach extended to 3D datasets, using the threshold size plug-in Brain Voyager QX (Forman et al., 1995). The minimum cluster size for the contrast Face > Scramble was 30 voxels. The minimum cluster size for the contrast Face > Words was 23 voxels. The minimum cluster size for the contrast Human voices > Natural sounds was 20 voxels.

###### Whole-brain parametric general linear model analyses

To find regions showing modulated response to faces due to face novelty and change in orientation, we conducted a whole-brain parametric analysis. Parametric modulation analysis is used to identify modulations in brain activation in response to consistent variations in stimuli belonging to the same group of objects (Büchel et al., 1998; Wood et al., 2008; Eck et al., 2013).

Each of the four conditions (trained faces, inverted faces, new faces, and scrambled faces) was assigned a predictor value used as a regressor in GLM analysis. All predictors were convoluted with a typical hemodynamic response function (Friston et al., 1999). This analysis was carried out twice. First, we used ordinal weights based on objective experience with the specific stimulus type: trained faces, followed by inverted and untrained faces (both untrained), followed by scrambled faces as a control. Second, weights were assigned to match the ratio of activation as reported in the literature for sighted individuals who perceive faces visually. The ratio of BOLD signal change between familiar faces and scrambled faces was calculated from Kanwisher et al. (1997) to be 3.2. The ratio of BOLD signal change between faces and inverted faces was calculated from Yovel and Kanwisher (2005) to be 1.23. The predictor weight for new faces received the same value as inverted faces in both analyses, following the hypothesis that strength of activation for novel faces will follow experience-related predictions.

To obtain regions showing not only face-related modulations but also differential activations in relation to change in orientation and face familiarity, we applied a conjunction (AND) condition, comprising the parametrically modulated condition and the “main” unmodulated face activation.

All parametric analyses were reported using a threshold of *p* < 0.005 before correction (Eck et al., 2013) (see Supplementary Figure 3A for results with *p* < 0.001), and then corrected for multiple comparisons using a cluster-size threshold adjustment for the Monte Carlo simulation approach extended to 3D datasets, using the threshold size plug-in Brain Voyager QX at *p* < 0.05 (Forman et al., 1995). The minimum cluster size for the parametric modulation using ordinal weights based on objective experience was 59 voxels, and the minimum cluster size for the analysis using literature-based weights was 57 voxels.

###### Single-subject fusiform gyrus peaks

To assess the individual variability of fusiform gyrus recruitment for auditory SSD face processing, and to ensure the peak of the activation in the ventral visual stream was indeed in the fusiform gyrus, we extracted the peak activation of each participant in the ventral visual stream, bilaterally for the contrast Trained faces > Scrambled faces. For demonstration purposes, a 6 mm sphere was created around these peaks and then plotted on a 3D graph. Results were also projected on a 3D brain.

###### Region of interest analysis

First, to investigate the preference for auditory faces over scrambled faces within the location of the right FFA in sighted individuals, we created a 6 mm sphere ROI around its canonical coordinates (Talairach coordinates: 40, −55, −10 (Kanwisher et al., 1997). Activation parameter estimates and *t*-values were sampled from this ROI in a group-level random-effects analysis. Second, to further investigate the results we obtained from the category experiments, namely to clarify the properties of activation in our FFA-like face-responding cluster in face versus other visual categories, we created a 6 mm sphere ROI around the peak of maximal overlap in the right fusiform gyrus in the probabilistic map obtained from the contrast Faces > Baseline, computed from the block design experiment on face-related modulations. This peak represented 100% overlap for face soundscapes, meaning that all participants showed activation for faces in this location (see Supplementary Figure 4B). Within this cluster, we extracted the average beta value per condition (faces, words, hand gestures). We first calculated the individual average activation for each of the three categories and then averaged them across the group, over both block and event-related experiments containing the three visual categories.

###### ANCOVA model

To explore the relationship between neural response and behavioral performance, we performed a whole-brain ANCOVA model. An average beta-value map for each participant resulting from the contrast upright faces > baseline across all face blocks was created. Beta values were then correlated with the corresponding individual behavioral identification performance for upright faces (average of percent correct across blocks). Due to technical issues resulting in loss of behavioral data from one participant, leading to a low number of participants in the analysis, results are reported uncorrected for multiple comparisons with *p* < 0.05.

#### Visualization of the results

For representational purposes, cortical reconstruction included the segmentation of the white matter using a grow-region function embedded in the Brain Voyager QX 2.0.8 software package. The cortical surface was then inflated. Group results were superimposed on a 3D cortical reconstruction of a Talairach normalized brain (Talairach and Tournoux, 1988).

## Results

### Face preferences in fusiform gyrus and its modulation by face properties

First, we analyzed the results of the block-design experiment containing blocks of trained cartoon faces, scrambled faces, untrained inverted faces, and entirely new faces (Supplementary Figure 1). fMRI results revealed face-shape preference in the fusiform gyrus (FG) largely resembling typical face processing as documented in the sighted. First, as shown in Figure 1B, we investigated the cross-subject overlap probability map created from the individual activation maps for the contrast Trained faces > Scrambled faces. This analysis aimed at assessing the anatomical consistency of activations across participants, and revealed bilateral FG activations across all individual subjects (Figure 1B), with activation peaks located lateral to the mid fusiform sulcus (Figure 1C). In addition, this analysis also revealed across-participants anatomically consistent bilateral clusters of activation in a region within the middle occipital gyrus, including the location of OFA as identified in the sighted, i.e., a core region of the face network, and a cluster of activation in the left IFG, another region implicated in face processing (Figure 1B). We also tested the same contrast again, this time using whole-brain RFX-GLM analysis. This result showed compatible results, namely, bilateral recruitment of FG and of the middle occipital sulcus, again including the location of OFA. Finally, to assess the dependency of our observed activations with the behavioral performance inside the scanner during the face identification task, we applied an ANCOVA model. Specifically, we conducted a whole-brain analysis correlating individual neural activation for upright-face blocks, with the individual success rate in upright-faces identification (see section “Materials and methods”). This analysis revealed a trend of stronger correlation between neural activation and behavioral performance in the fusiform gyrus only, in close proximity to the FG recruitment for the contrast of upright faces > scrambled faces (Supplementary Figure 4C).

Second, to explore the anatomical relationship between face-responsive activation in the blind and the face-responsive activation reported in the sighted, we conducted an ROI-GLM analysis using the canonical coordinates of the right FFA (see section “Materials and methods”; Talairach coordinates of ROI peak: 40, −55, −10). Results for the contrast Trained Faces > Scrambled Faces showed a significant recruitment for face soundscapes in our group of congenitally blind participants, within this defined ROI (Figure 1D).

**FIGURE 2:**
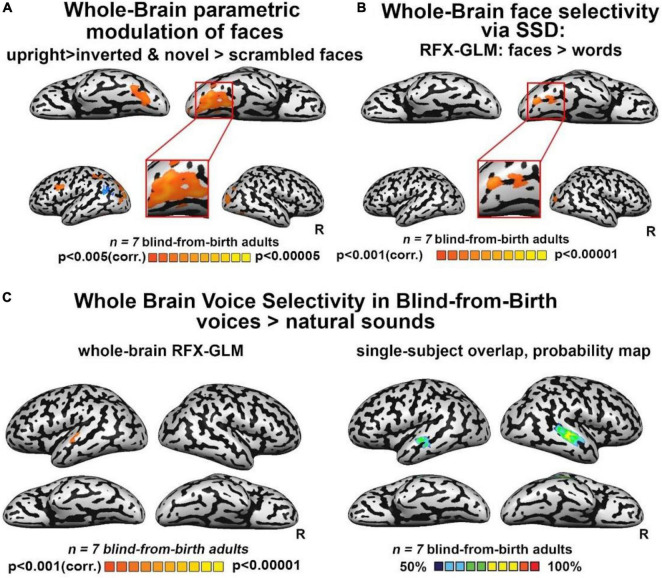
**(A)** Whole-brain parametric general linear model (GLM) shows that bilateral clusters in fusiform gyrus (FG) are maximally activated by trained faces, then by face orientation and face novelty, and last by scrambled faces. A similar modulation was observed also for occipital face area (OFA) and inferior frontal gyrus (IFG), two other cortical regions known to be involved in face processing. **(B)** Whole-brain RFX-GLM with the contrast Faces > Words shows recruitment of the right FG (Talairach coordinates of peak 31, –57, –16), together with a cluster in the right middle occipital gyrus, including OFA, another cortical region known to prefer face-related information. **(C)** Human voices > natural sounds. Left: RFX-GLM analysis shows the expected recruitment of the left superior temporal gyrus (Talairach coordinates: –60, –19, 10), compatible with the known location of the TVA but crucially, no recruitment of FG. Right: The probability map obtained from the overlap of single participants’ activations for the same contrast, reveals consistent activations in the bilateral TVA but crucially, no activation in the ventral visual stream nor in FG.

Third, we investigated the extent to which FFA-like activations are modulated by untrained changes in orientation (upright/inverted faces) or by face novelty (trained/entirely new faces) as in the sighted. Whole-brain analyses for the direct contrasts between these conditions did not yield fusiform gyrus activations. However, when using a more permissive threshold, we observed recruitment of the right FG for trained faces versus novel faces (Supplementary Figure 3E), while the contrast of upright trained faces over inverted faces did not show any significant activation in the fusiform gyrus. Interestingly, this latter contrast yielded a bilateral activation of the sub-insular cortex, a region that has been suggested to be involved in the processing of configural aspects of face perception in the sighted brain (see Supplementary Figures 3C,D,F, for the results of inverted and novel faces vs scrambled faces). Importantly, however, when we performed a whole-brain rank-order parametric analysis on these data, we observed a bilateral cluster peaking in the FG which was maximally activated by trained faces, followed by inverted and untrained faces, and then by scrambled faces – in other words, a modulation resembling face-preference properties reported in the sighted brain (Figure 2A). These results were replicated when we performed the same analysis using weights derived from the literature (Supplementary Figure 4A).

### Faces versus other categories

Next, we tested whether our reported FFA-like preference for face shapes was maintained when faces were contrasted with other object categories: words and hand gestures, also conveyed *via* SSD and trained in parallel to faces. As seen in Figure 2B, the whole-brain RFX-GLM analysis comparing activations for SSD-trained faces to activations for SSD-trained written words showed a cluster of significant preference for faces, with a whole-brain peak in the right FG. A second peak of activation was in an anatomical location near the location of the right OFA. Importantly, these results exclude the possibility that the FFA-like activations resulted mainly from acquired experience in perceiving shapes belonging to a novel set of stimuli.

In addition, the direct contrast of SSD-conveyed faces and hand gestures showed no face preference in FG. However, beta values for the three conditions (faces, words, hand gestures) obtained from an independent FFA-like cluster (see section “Materials and methods”), revealed a trend of activation: the FFA-compatible region was maximally activated by faces, then by hand gestures, and last by words (Supplementary Figure 4B). These results suggest that similarly to what has been reported for the sighted, the lateral posterior fusiform gyrus of congenitally blind adults responds to animate objects.

Finally, to characterize the response of our blind participants to human voices, we employed the seminal human-voice localizer protocol used to unravel voice specializations in the healthy population (see section “Materials and methods”). As seen in Figure 2C, mirroring what is typically documented in the sighted, whole-brain RFX-GLM analysis showed activations in the TVA for the contrast Voices > Natural sounds (see also Supplementary Figure 3B). Crucially, no voice preference emerged in FG or in any other location compatible with a known face-responsive region in the sighted. The cross-subject overlap probability map created from all the individual participants’ activations for the same contrast (Figure 2C) confirmed there were no anatomically consistent activations in the ventral stream of our congenitally blind participants for human voices. These results indicate that the activation in the visual cortex elicited by whole-face soundscapes is not due to high-level interpretation of person-specific information or human-generated sounds.

## Discussion

This study explored the properties of face-shape preference in the ventral visual stream of the congenitally blind, following tailored face training *via* the atypical auditory modality. Specifically, a group of SSD-expert congenitally blind adults learned to perceive cartooned faces in a ∼12 h unique training program aimed at conveying faces auditorily *via* soundscapes, in a shape, spatial layout, and color-preserving manner, using a visual-to-auditory SSD (Arbel et al., 2022). Following training, the ventral visual stream in the blind brain showed preference for faces versus scrambled sounds in the fusiform gyrus as well as a parametrically modulated preference for trained SSD-faces over inverted, novel, and scrambled faces, also peaking in the fusiform gyrus. The same preference also emerged when faces were contrasted with stimuli belonging to another category of newly learned SSD-stimuli (reading words composed of letters from an entirely new alphabet), even though both categories were trained for a comparable time (Figure 2B). In contrast to the visual cortex, no modulation of the auditory cortex was observed in any of the analyses suggesting that participants attended all stimuli similarly. Importantly, activations in all the aforementioned analyses, also included a cluster within the middle occipital gyrus, including the location of the sighted OFA, namely, another region of the core face network. This suggests that acquired experience in recognizing exemplars of stimuli belonging to a novel category of objects cannot entirely explain the pattern of reported FG activation.

In addition, in contrast to the preference of faces over words, the ventral visual stream in the blind brain showed no preference for faces over hand gestures. Hand gestures represent another animate category of objects for which, like faces, blind people have little perceptual experience through their remaining sensory modalities (although they can access them to some extent *via* proprioceptive cues). While the direct contrast between trained faces and hand gestures did not yield any preference within the ventral visual stream, ROI analyses showed a trend for a preference in the blind right ventral stream for faces, as faces activated it the most, followed by hand gestures and words (Supplementary Figure 3G), similarly to the haptic domain (Murty et al., 2020). These results fit well with accumulating evidence on the sighted brain suggesting that bodies and faces share brain representations in the fusiform gyrus, thus allowing the building of a unified whole-person representation, leading to the perception of naturalistic stimuli (Kaiser et al., 2014; Fisher and Freiwald, 2015). Our results indicate the same process might be preserved and at work in the blind brain, thereby suggesting that such integrated body-faces representations are sensory-independent. Moreover, our results highlight that their emergence is not constrained to the exposure to visual inputs early in life or across the lifespan. Future studies may further investigate this intriguing conclusion. For instance, they might investigate through MVPA whether body-responsive and face-responsive voxels within this region are dissociable in congenitally blind adults, as has been demonstrated in the sighted (Kim et al., 2014). Our results strengthen the initial conclusions already suggesting that animate and not only inanimate object representations are retained in the blind ventral “visual” stream (Bi et al., 2016; Murty et al., 2020), with properties largely resembling typical specializations observed in the sighted brain.

Finally, we found no recruitment in the fusiform gyrus or in any other location compatible with a known face-responsive region in the sighted brain, to human voices, the main person-identification method used by blind adults in everyday life (Figure 2C). These results excludes the possibility that the observed FG activations were driven by general person-specific associations rather than by the processing of whole-face SSD-conveyed shapes and are in line with previous results (Hölig et al., 2014; Dormal et al., 2018).

Taken together, these results strengthen the emerging notion that brain specializations are driven by predispositions to process sensory-independent computations rather than unisensory-specific inputs, as classically conceived (James et al., 2002; Amedi et al., 2007, 2017; Ricciardi et al., 2007; Kupers et al., 2010; Collignon et al., 2011; Reich et al., 2011; Wolbers et al., 2011; Ptito et al., 2012; Striem-Amit et al., 2012; Striem-Amit and Amedi, 2014). It has recently been proposed that this computation-selective and sensory-independent organization originates from two non-mutual exclusive principles: (1) local tuning to sensory-independent task/computation distinctive features (e.g., a predisposition of FFA to process sensory-independent face-distinctive shapes); (2) preserved network connectivity (e.g., preserved connection between FFA and the rest of the face-network) (Hannagan et al., 2015; Heimler et al., 2015; Amedi et al., 2017).

Furthermore, the results argue against some aspects of the classic assumptions that stem from the seminal studies by Hubel and Wiesel (Hubel and Wiesel, 1962; Wiesel and Hubel, 1963, 1965) positing that the key driver of the emergence of typical brain specializations is the exposure to unisensory-specific inputs during early infancy, i.e., during specific time windows termed “critical/sensitive-periods” when the brain is particularly plastic (Knudsen, 2004). Indeed, our findings suggest that the potential to process specific computations in specific cortical regions may not vanish with the closure of critical periods but might be re-awakened at any time across the lifespan, if computation-tailored training is provided (Heimler and Amedi, 2020). Specifically, the current results are compatible with the conclusion that our tailored SSD training may have guided/facilitated the recruitment of typical face regions in the deprived visual brain *via* auditory inputs by relying on the two non-mutually exclusive – and possibly hard-wired – principles proposed above as underlying the emergence of computation-selective and sensory-independent cortical organization. In the case of face processing, the existence of hard-wired face-selective regions is supported by studies of infants showing face preference and discrimination abilities at birth (Turati et al., 2006), together with hard-wired connectivity within the face-related network (Buiatti et al., 2019; Kamps et al., 2020), possibly even suggesting a genetic component of face-related processing, as highlighted by studies with homozygote twins (Polk et al., 2007; Wilmer et al., 2010). The connectivity properties of the whole face network still need to be explored in congenitally blind adults, as do the connections after dedicated face training (for evidence of preserved network-connectivity for other SSD-trained visual categories, see Abboud et al., 2014; Striem-Amit and Amedi, 2014. Another open issue concerns the pathway through which auditory information reaches the deprived visual cortex. Future studies should disentangle whether this is mediated by top-down connections from higher-order regions or *via* direct audio-visual connections (Collignon et al., 2013; Sigalov et al., 2016), or by bottom-up projections from sub-cortical regions (Müller et al., 2019).

Importantly, these results also carry implications for sensory recovery. While some studies on visually restored patients have reported lack of face selectivity even years after sight recovery (Fine et al., 2003; Röder et al., 2013; Grady et al., 2014), none has provided patients with a tailored, computation-oriented training aimed at teaching face-shape recognition.

Evidence in favor of the beneficial role of (multisensory) computation-specific training in sensory recovery is starting to emerge (i.e., pairing the restored sense, e.g., vision, with a familiar one, and audition). This evidence, mostly coming from animals, documents more efficient computation-selective neural recruitment in sensory restored individuals who have undergone multisensory computation-oriented training (DeGutis et al. (2007) and Isaiah et al. (2014); for comparable results for partial deprivation, see Jiang et al., 2015). This approach might be a promising rehabilitative venue to further tune face classification abilities developed *via* natural experience in visually restored patients (Gandhi et al., 2017). We suggest that such multisensory training approach might maximize the restoration outcomes, as the familiar sense (e.g., audition) might guide the restored sense (e.g., vision) to recruit its typical sensory cortex by promoting a network adaptability process (Heimler et al., 2015; Heimler and Amedi, 2020; Maimon et al., 2022). Future studies may more systematically investigate this intriguing hypothesis and track the extent to which such types of training might indeed aid the (re) establishment of typical cortical recruitment by the restored visual input, in line with the predictions of the computational-selective cortical organization.

While some face preference activations observed in our congenitally blind participants were largely similar to typical results in the sighted population, there were also some differences. First, preference in the fusiform gyrus was bilateral rather than right-lateralized as classically reported (Kanwisher et al., 1997; Figure 1). A possible explanation of this difference is our congenitally blind participants’ lack of experience in the processing of whole faces *via* audition (∼12 h SSD training), i.e., we cannot exclude that with additional experience the activation will become right lateralized. Another possibility is that these bilateral FG activations might relate to the reduced left-lateralization for language repeatedly documented in the blind population (Lane et al., 2015, 2017; Pant et al., 2020). Indeed, previous studies showed reduced face-related lateralization in other populations with reduced language-lateralization, such as ambidextrous or left-handed individuals (Badzakova-Trajkov et al., 2010; Willems et al., 2010; Bukowski et al., 2013; Dundas et al., 2015; Gerrits et al., 2019). Specifically, several accounts currently propose that face and language processing compete for the same representational space in the human brain, and faces become right lateralized as a consequence of left regions being recruited by reading due to proximity to the rest of the language areas (Dundas et al., 2015).

Finally, our results showed preserved face preference in the blind brain not only in the fusiform gyrus, but also in other regions known to belong to the face network. Specifically, we showed consistent activations in all our main contrasts in the middle occipital gyrus, including a region compatible with OFA (Figures 1B, 2A,B), which is a core region of the face network and it is described as involved in the perceptual processing of facial features (Kanwisher et al., 1997; Gauthier et al., 2000b; Rossion et al., 2003; Pitcher et al., 2011). Further investigations into the roles of the FFA and OFA in people who are congenitally blind are still required to assess more systematically the similarities as well as the potential differences between blind and sighted brains regarding specific properties of both nodes. For instance, the role of both regions in the identification of complete face-images (i.e., holistic processing) vs. facial features (i.e., parts-based processing), as evidenced by the increased sensitivity documented in the sighted brain for the scrambling of faces in the FFA compared with OFA (Lerner et al., 2001). Future studies should also explore aspects directly related to cognitive processes mediating auditory face identification *via* sweep-line algorithms such as the present SSD, namely whether these processes involve object-based mechanisms or rather approaches more specific to visual faces perception such as holistic face processing and the extent to which specific characteristics of SSD-mediated processing influence recruitment of face-related regions within the face-network.

In contrast to observed recruitment of the OFA, we did not observe any activation in the STS another core face region, which is known to process the changeable aspects of faces, such as expressions (Phillips et al., 1997), direction of eye-gaze, and lip movements (Puce et al., 1998; Allison et al., 2000; Haxby et al., 2000; Lahnakoski et al., 2012; Zhen et al., 2015). Notably, however, these computations were not part of our training program nor where taken into consideration in the behavioral tasks performed by our participants in the scanner. Future studies addressing these specific aspects of face perception may provide crucial insight into possible constraints of visual experience on the development of face-specific cortical regions and on the face network as a whole.

While the present work investigated auditory face perception in congenitally blind for the first time, the novelty of the chosen stimuli imposes some limitations on the experimental design. First, due to the presentation of novel images during the experiment, participants could not perform the exact same identification task for all presented stimuli. Second, while we investigated neural responses following extensive face training, we are unable to attest to the role of the fusiform gyrus and the rest of the face-identification network in congenitally blind, prior to any face training. Future investigations could further explore the effect of training on the engagement of the face-processing network, the potential role of “face imagination” or the abstract representation congenitally blind have of faces prior to face training, as well as further balance task and task-free designs to further define the properties of the neural face network in the congenital absence of vision.

Taken together, our results show that the ventral visual stream of congenitally blind adults can be recruited by face processing *via* non-traditional, non-visual, sensory information acquired during adulthood, and retain some of its properties despite life-long visual deprivation. Our results provide evidence supporting the presence of a predisposition for sensory-independent and computation-specific processing in the human brain. We show that this predisposition is retained even in life-long sensory deprivation when a given category of stimuli, face-shapes in the current case, has been largely inaccessible across the lifespan through the available sensory inputs. Our results thus have implications for visual recovery by suggesting that such predispositions can be (re) awakened in adulthood if tailored computation-oriented training is provided.

## Data availability statement

The raw data supporting the conclusions of this article will be made available upon request to the authors, without undue reservation.

## Ethics statement

The studies involving human participants were reviewed and approved by the Hadassah Medical Center Ethics Committee and the Hadassah Helsinki Committee. The patients/participants provided their written informed consent to participate in this study.

## Author contributions

RA and AA conceived and designed the experiments. RA performed the experiments. AA and BH contributed to the data analysis supervision and guidance and analyzed the data. RA, BH, and AA wrote the manuscript. All authors contributed to the article and approved the submitted version.
