# Supplementary material for: Face shape processing via visual-to-auditory sensory substitution activates regions within the face processing networks in the absence of visual experience
Source: Front Neurosci. 2022 Oct 3;16:921321. doi: 10.3389/fnins.2022.921321 (PMC9576157; doi:10.3389/fnins.2022.921321)
Supplement: Supplementary file 1 [file Data_Sheet_1.docx]

**Supplementary information**


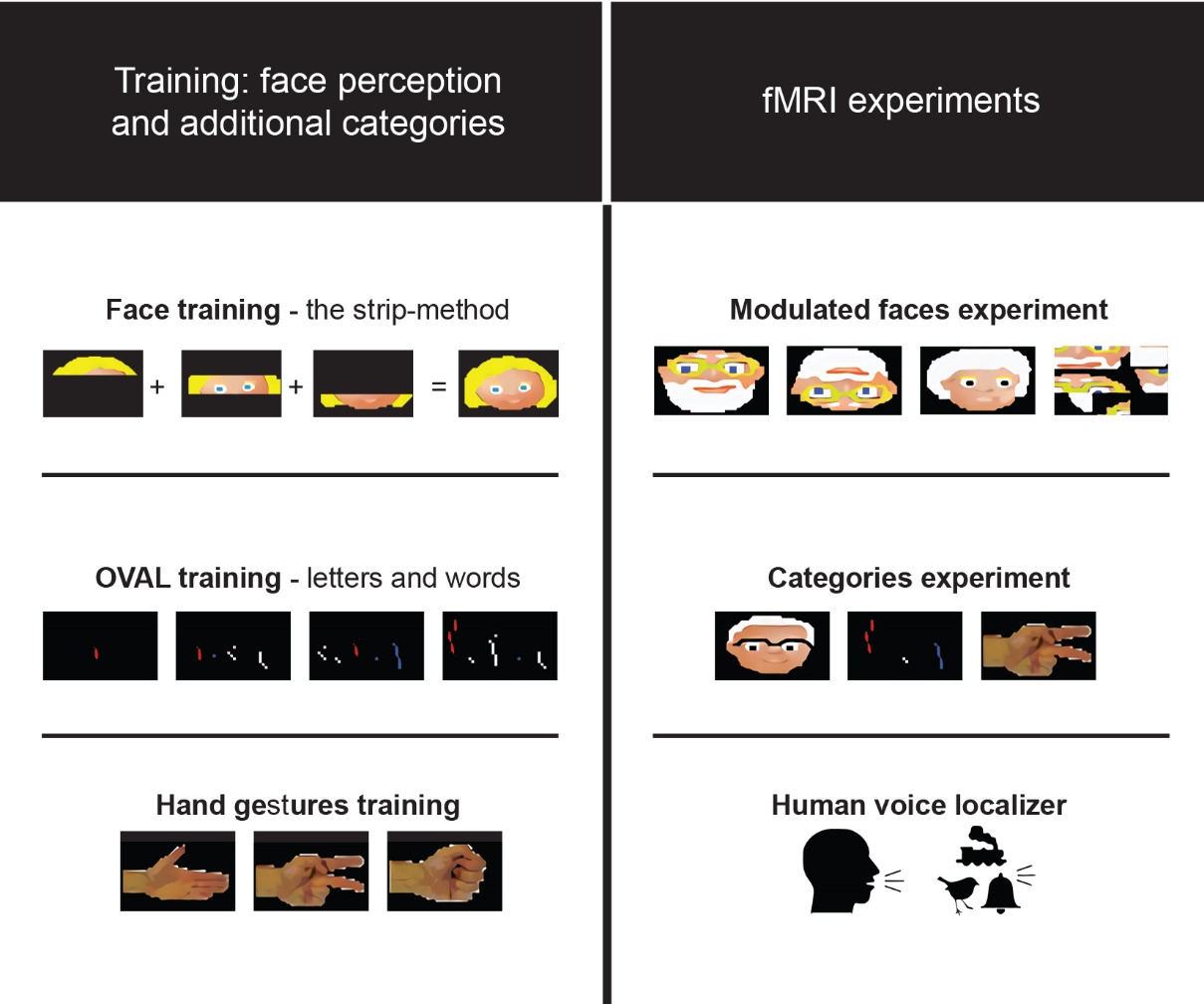


Supplementary Figure 1 (S1): Experimental paradigm. Participants were trained on face recognition via the auditory modality, as well as on word reading and hand gestures. Following training, they participated in several imaging experiments designed to investigate the neural face recognition system in congenitally blind.

**­**

**
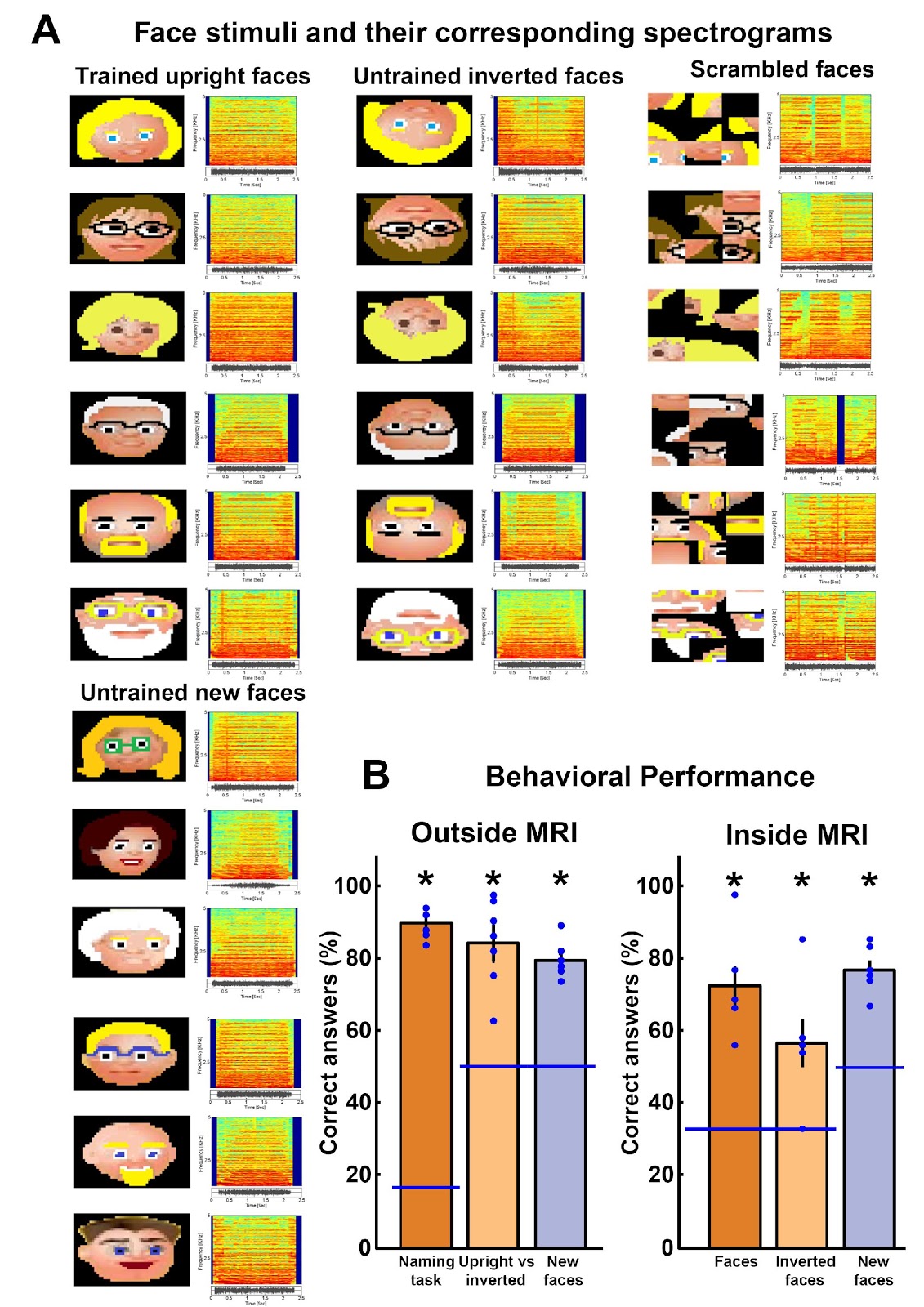
**

Supplementary Figure 2 (S2): S2A. Face stimuli used in the block-design fMRI experiment. In each panel, the visual image of each specific face is depicted, together with the resulting spectrogram after the EyeMusic conversion. S2B. Behavioral results of participants inside (left) and outside the scanner (right). Blue dots represent performance of single participants. Blue solid lines represent chance level for each task. Asterisks represent significance against chance level. Error bars depict standard deviation.


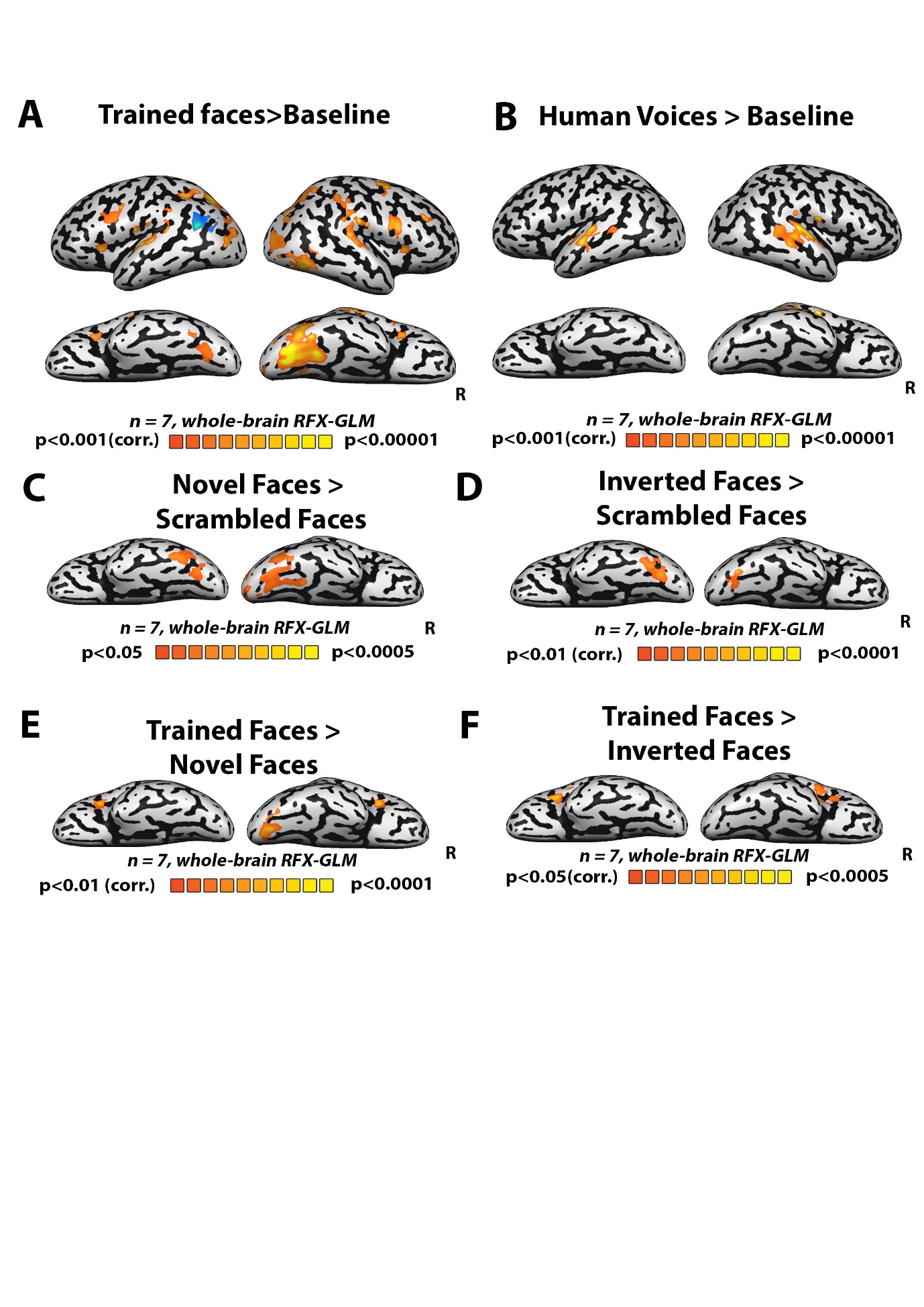


Supplementary Figure 3 (S3). S3A. Whole-brain RFX-GLM for the contrast Trained faces>Baseline. Activations are observed both in auditory and visual cortices, with strong and extensive activations of the right ventral visual stream, with a peak in the fusiform gyrus (FG) lateral to the mid-fusiform sulcus, along with a smaller cluster of activation also in the left fusiform-gyrus. S3B. Whole-brain RFX-GLM with the contrast Human voices>Baseline. S3C. Whole-brain RFX-GLM with the contrast Novel faces>Scrambled faces. This contrast reveals bilateral fusiform gyrus activations with a threshold of p<0.05, uncorrected for multiple comparisons. S3D. A whole-brain RFX-GLM with the contrast Inverted faces>Scrambled faces shows bilateral recruitment of the fusiform gyrus. S3E. Trained faces > Novel Faces: Activation in the right fusiform gyrus (FG) is observed only when increasing the threshold to p<0.01, before correction for multiple comparisons. S3F. A whole-brain RFX-GLM with contrast Upright faces (trained)>Inverted faces (untrained) does not show selectivity in the fusiform gyrus. However, we observe recruitment of the sub-insular cortex, a region suggested to be involved in configural processing of faces.


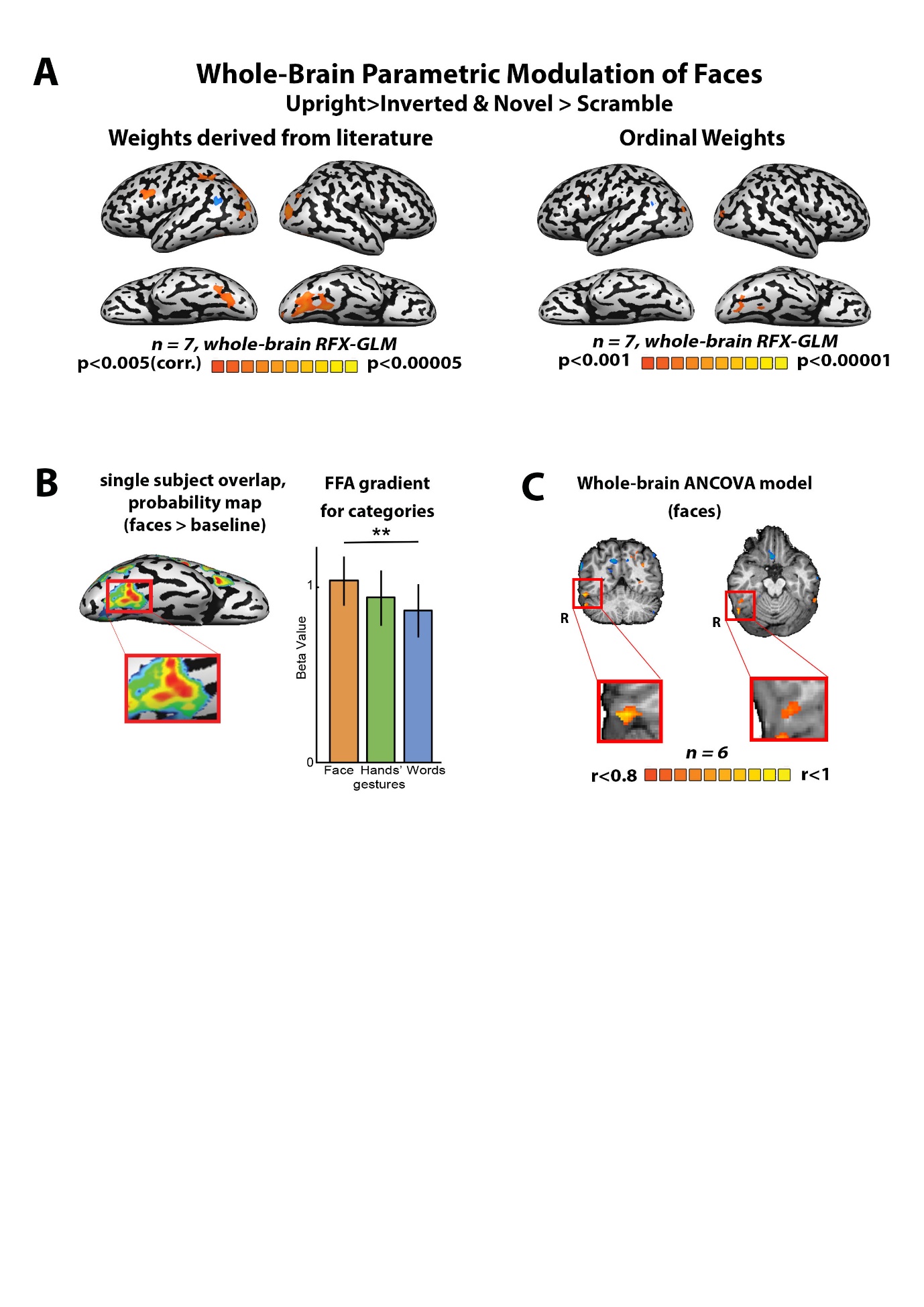


Supplementary Figure 4 (S4). S4A. Whole-brain parametric modulation for faces, in relation to Fig. 2A. Here we show a replication of the analysis using weights derived from the literature (see Methods) (left panel) and the uncorrected results using a stricter threshold (p<0.001) with the same ordinal weights as in Fig. 2A (right panel). S4B. Ventral view of the results from the cross-subject overlap-probability map with contrast Trained faces>Baseline calculated from the results obtained in the block-design experiment for face modulation.  The peak with maximal overlap (100%) across participants (i.e., all participants activated those voxels for this contrast) is in the right fusiform gyrus (FG) and is used as seed for the extraction of beta values from the visual categories experiment (left). Results of the ROI analyses in the right FG for the visual categories experiment: a gradient of activation is revealed such that SSD-conveyed faces activate this region the most, followed by SSD-conveyed hand gestures, and then by SSD-conveyed words (right). S4C. A whole-brain ANCOVA model, correlating beta values for upright-faces blocks with behavioral performance for auditory upright-faces identification. Whole-brain analysis shows cluster of significant correlation in the right Fusiform Gyrus.

Table 1: Activation peaks and sub-peaks for auditory faces vs. scramble

| Hemisphere | Peak Anatomical Location | Talairach peak coordinates | | | Peak  T-value | Peak p-value | Cluster size (full ROI) |
| --- | --- | --- | --- | --- | --- | --- | --- |
|  |  | x | y | z |  |  |  |
| left | Inferior Frontal Gyrus | -45 | 8 | 31 | 8.23 | 0.0002 | 906 |
|  | Middle Occipital Gyrus | -27 | -85 | 16 | 10.89 | 0.00004 | 4342 |
|  | Fusiform Gyrus | -34 | -65 | -14 | 7.66 | 0.0003 |  |
|  | Middle Occipital Ggyrus | -33 | -82 | -8 | 7.78 | 0.0002 |  |
|  | Middle Occipital Gyrus | -27 | -84 | 19 | 8.06 | 0.0002 |  |
| right | Fusiform Gyrus | 19 | -68 | -13 | 7.93 | 0.0002 | 1097 |
|  | Medial Occipito-temporal Gyrus | 21 | -94 | -5 | 8.36 | 0.0002 | 3979 |

Table 3: Activation peaks and sub-peaks for auditory faces vs. words

| Hemisphere | Peak Anatomical Location | Talairach peak coordinates | | | Peak  T-value | Peak p-value | Cluster size (full ROI) |
| --- | --- | --- | --- | --- | --- | --- | --- |
|  |  | x | y | z |  |  |  |
| right | Middle Occipital Gyrus | 30 | -75 | -4 | 11.31 | 0.00004 | 3585 |
|  | Fusiform Gyrus | 31 | -57 | -16 | 8.81 | 0.0001 |  |
|  | Middle Occipital Gyrus | 29 | -75 | -7 | 11.31 | 0.00003 |  |
|  | Middle Occipital Gyrus | 30 | -80 | 11 | 8.6883 | 0.0001 |  |
|  | Cuneus | 15 | -85 | 11 | 7.82179 | 0.0002 |  |

Table 4: Activation peaks for human voices vs. natural sounds

| Hemisphere | Peak Anatomical Location | Talairach peak coordinates | | | Peak  T-value | Peak p-value | Cluster size (full ROI) |
| --- | --- | --- | --- | --- | --- | --- | --- |
|  |  | x | y | z |  |  |  |
| right | Transverse Teomporal Gyrus | -60 | -19 | 10 | 11.61 | 0.00003 | 1286 |

Table 5: Activation peaks for parametric modulation

| Hemisphere | Peak Anatomical Location | Talairach peak coordinates | | | Peak  T-value | Peak p-value | Cluster size (full ROI) |
| --- | --- | --- | --- | --- | --- | --- | --- |
|  |  | x | y | z |  |  |  |
| right | Middle Occipital Gyrus | 24 | -91 | -5 | 7.3 | 0.0003 | 17666 |
|  | Fusiform Gyrus | 23 | -45 | -15 | 6.73 | 0.0005 |  |
|  | Fusiform gyrus | 27 | -68 | -12 | 6.82 | 0.0005 |  |
|  | Middle Occipital Gyrus | 26 | -86 | -3 | 7.3 | 0.0003 |  |
|  | Middle Occipital Gyrus | 29 | -78 | 14 | 6.99 | 0.0004 |  |
|  | Cuneus | 10 | -85 | 14 | 6.14 | 0.0009 |  |
| left | Middle Occipital Gyrus | -27 | -85 | 16 | 7.98 | 0.0002 | 13291 |
|  | Fusiform Gyrus | -32 | -66 | -12 | 5.81155 | 0.001 |  |
|  | Precuneus | -25 | -66 | 31 | 6.19 | 0.000869 |  |
|  | Middle Occipital Gyrus | -26 | -84 | 19 | 7.98 | 0.0002 |  |
|  | Middle Occipital Gyrus | -15 | -92 | 12 | 7.44 | 0.0003 |  |
|  | Precuneus | -27 | -46 | 40 | 6.77 | 0.0005 | 1990 |
|  | Inferior Frontal Gyrus | -42 | 5 | 28 | 6.21 | 0.0008 | 1927 |
